# Fatal CTLA‐4 heterozygosity with autoimmunity and recurrent infections: a de novo mutation

**DOI:** 10.1002/ccr3.1257

**Published:** 2017-11-06

**Authors:** Maria Francisca Moraes‐Fontes, Amy P. Hsu, Iris Caramalho, Catarina Martins, Ana Carolina Araújo, Filipa Lourenço, Anna V. Taulaigo, Ana Lladó, Steven M. Holland, Gulbu Uzel

**Affiliations:** ^1^ Unidade de Doenças Auto‐imunes Serviço Medicina 7.2 Hospital de Curry Cabral Centro Hospitalar de Lisboa Central Lisboa Portugal; ^2^ Instituto Gulbenkian de Ciência Oeiras Portugal; ^3^ Laboratory of Clinical Infectious Diseases National Institute of Allergy and Infectious Diseases Bethesda Maryland USA; ^4^ CEDOC, Chronic Diseases Research Center Immunology, NOVA Medical School/FCM Universidade Nova de Lisboa Lisbon Portugal

**Keywords:** CTLA‐4 mutation, Evans syndrome, hypogammaglobulinemia, sepsis

## Abstract

Primary immunodeficiency disorders are rarely diagnosed in adults but must be considered in the differential diagnosis of combined recurrent infections and autoimmune disease. We describe a patient with CTLA‐4 haploinsufficiency and an abnormal regulatory T‐cell phenotype. Unusually, infections were more severe than autoimmunity, illustrating therapeutic challenges in disease course.

## Introduction

We report the identification of a fatal heterozygous mutation in CTLA‐4. The mutation was predicted to decrease protein stability resulting in haploinsufficiency and decreased CTLA‐4 expression in CD4+Foxp3+ T cells. The patient suffered from autoimmunity (Evan's syndrome), lymphoproliferation (diarrhea with intestinal inflammation), and immunocompromise (severe infections).

Heterozygous cytotoxic T lymphocyte antigen‐4 (CTLA‐4) mutations have recently been recognized to cause diverse clinical phenotypes 1, 2 with unpredictable responses to a wide range of therapies, ranging from immunosuppressants to bone marrow transplantation 3. We present a patient with a novel mutation and a fatal outcome.

## Case Report

In 2013, a 45‐year‐old Portuguese man (born to nonconsanguineous parents) was admitted for sepsis. An elective total hip replacement 6 months before had been followed by recurrent urosepsis. Clinical features and laboratory investigations are presented in Table 1. Evans syndrome had been diagnosed at 9 years, at which time lifelong steroid therapy was started. He had multiple episodes of otitis during childhood. From young adulthood, he complained of intermittent diarrhea. Previous upper and lower gastrointestinal endoscopic studies failed to reveal any abnormality except for a mild ileal inflammatory infiltrate on histology. He had been hospitalized three separate times over the past 10 years for episodes of hemolysis and severe thrombocytopenia, pneumonia, and lower limb cellulitis. There was no relevant family history.

**Table 1:** Patient's features, investigation results, and immunological studies up to the diagnosis

Clinical features—age (y) and treatment from diagnosis	Immunoglobulins and lymphocyte counts
9 y and childhood Evans syndrome Multiple episodes otitis 25–42 y Episodic diarrhea Multiple hospital admissions: hemolysis + thrombocytopenia pneumonia, lower limb cellulitis Cholecystitis Bilateral osteonecrosis of femoral head and condyles 43 y Elective right hip replacement. Postoperative: recurrent urosepsis caused by *Enteroccocus faecalis*,* Klebsiella pneumoniae* and *Pseudomonas aeruginosa*. 44 y—October 2013 Facial vitiligo, hepatosplenomegaly Hemoglobin 12.5 g/L; leukocytes: 8700/*μ*L; platelets 28000/*μ*L Benign prostatic hypertrophy; urethral stricture; trabeculated bladder Duodenal, ileal and bladder biopsy: inflammatory infiltrate (not characterized) Negative: direct Coombs, ANA, EBV DNA, CMV DNA, hepatitis B, C, HIV, proteinuria, urinary Ig loss Antiplatelet Ab positive	44 y – Pre‐IVIG – October 2013 Immunoglobulins (g/L) (normal range) IgG **3.4** (6.9–14.0); IgA **0.4** (0.88–4.1); IgM 0.77 (0.34–2.1); IgE <0.4 (<10); IgD <0.01 (0.01–1.5) Lymphocytes: **680**/*μ*L (normal >1000/*μ*L) Subpopulations, [%; total cells/*μ*L (normal range)]: *T cells*: CD3^+^ 86% (55–84); 608 (690–2540) CD4^+^ 75% (31–60); 528 (410–1590) CD8^+^ **11**% (13–41); **80** (190–1140) *B cells*: CD19^+^ **2**% (6–25); **17** (90–660) *NK cells*: CD3^−^/CD16^+^56^+^ 11% (5–27); **78** (90–590) Maturation and activation markers [%; total cells/*μ*L (normal range)]: *CD4* ^*+*^ *T cells*: CD45RA^+^ 13% (3–59); **24** (27–833) CD45RO^+^ **70%** (15–69); **130** (167–670) *CD8* ^*+*^ *T cells*: CD45RA^+^ 50% (6–84); 38 (19–508) CD45RO^+^ **66%** (4–49); 51 (15–275) *B‐cell subpopulations gated out of CD19* ^*+*^ : Naive CD27^−^ IgD^+^ IgM^+^ 64% (42–82); **4** Memory CD27^+^ 30%; **2** Memory CD27^+^ IgD^+^ Marginal zone 24%; **6** Memory switched CD27^+^ IgD^−^ **1** Transitional CD38^+^ IgM^hi^ **0.1**

ANA, antinuclear antibody; IVIG, intravenous immunoglobulin substitution; MP, methyl–prednisolone (500 mg/day × 3); Ab, antibody; values outside the normal range are given in bold.

Clinical examination was unremarkable apart from proportionate short stature (150.0 cm), hepatosplenomegaly but no lymphadenopathy. He had a normal hemoglobin and platelet count. Despite a normal total leukocyte count, there was lymphopenia. Flow cytometric analysis performed while patient was under prednisolone 5 mg/day showed a marked reduction in CD8^+^ T cells and CD19^+^ B cells. Total IgG and IgA were low, IgE and IgD were undetectable, and IgM was normal. Antidiphtheria Ab: 0.44 UI/mL (protection titer >1.0 UI/mL); peripheral blood mononuclear cell proliferation to PHA, PPD, and Candida were slightly reduced. He also had prostatic hypertrophy, a urethral stricture, and a trabeculated bladder. He was discharged on intravenous immunoglobulin (IVIG) (0.6 g/kg body weight every 3 to 4 weeks) and maintained on prednisolone 5 mg/day, prophylactic nitrofurantoin 100 mg/day, and tamsulosin 0.4 mg/day.

**Figure 1:**
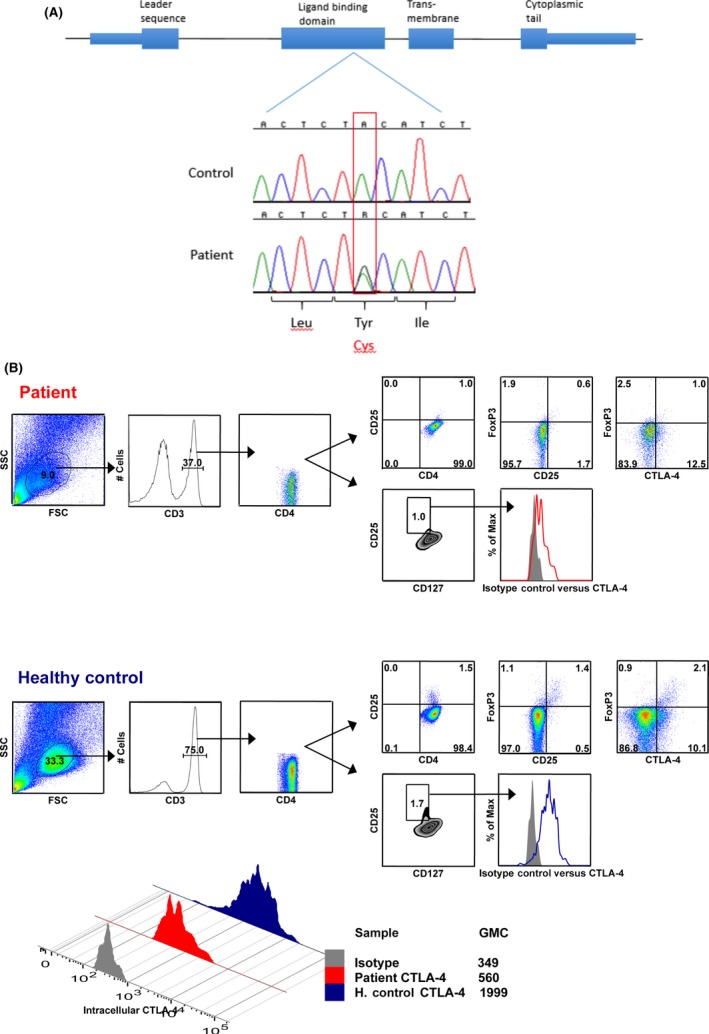
(A) Diagnosis of the CTLA‐4 mutation. Broad genetic screening using a custom panel of many immune‐related genes using an ion proton next‐generation sequencer, followed by Sanger sequencing, was performed at the Laboratory of Clinical and Infectious Diseases of the National Institute of Allergy and Infectious Diseases, Bethesda, Maryland. This figure shows Sanger sequencing confirmation of the mutation. The top tracing is from a control and the bottom tracing from the patient. CTLA‐4 sequencing was performed after amplification of the four exons. Shown is the translated protein of the gene containing 4 domains, indicated above the exons. In the patient, two peaks representing the wild‐type allele (containing A, like in the control) and the mutated allele (containing the mutant G) are shown. This mutation caused a substitution of cysteine (Cys) for tyrosine (Tyr) in the ligand‐binding domain (exon 2). (B) CTLA‐4 expression within regulatory T cells. Methods for surface staining of peripheral blood mononuclear cells (PBMCs) using validated antibodies and intracellular staining with anti‐FoxP3 and CD152 antibodies, sample acquisition, and analysis were previously described. Flow cytometric analysis of FoxP3, CD25, and CTLA4 expressions within gated CD4^+^ T peripheral blood cells (defined as CD4^+^ CD3^+^ cells) from the patient and a healthy control. Also depicted in histogram overlay is the expression of CTLA4 and corresponding isotype control within CD4^+^ CD25^+^ CD127^hi^ cells. For direct comparison of CTLA4 expression within CD4^+^ CD25^+^ CD127^hi^ cells from patient and healthy control, a histogram overlay with the corresponding values of CTLA4 geometric mean is shown in below panel. Foxp3 and CTLA‐4 stainings are intracellular and performed in the absence of stimulation.

Broad genetic panel screening followed by Sanger confirmation identified the heterozygous CTLA‐4 mutation c.380A>G, p.Tyr127Cys (Figure 1A). This mutation was not found in ESP6500 4, dbSNP 5, 1000G 6, or ExAC 7 databases. Computational predictors (SIFT 8, Polyphen‐2 9, MutPred 10, PredictSNP 11, PROVEAN 12, PON‐P2 13, Mutation Taster 14, MetaSNP 15, I‐Mutant Suite 16, and IStable 17) of the effect of missense mutations assessed Tyr127Cys to be deleterious, damaging, pathogenic, and likely disease causing or to decrease protein stability (Table 2). No familial segregation analysis could be performed as the patient′s first‐degree relatives (reportedly healthy) refused genetic testing, and the patient had no progeny. Further immunophenotyping disclosed excess in the frequency of mature T cells, contrasting with absence of memory B lymphocytes. Within the CD4^+^ T lymphocytes, the frequency of the Foxp3^+^ regulatory T‐cell (Treg) population was slightly reduced compared to a healthy control. CD4^+^Foxp3^+^ T cells additionally displayed reduced levels of CD25 and CTLA‐4 expressions (Figure 1B).

**Table 2:** Computational predictor results for the substitution of a tyrosine for a cysteine at amino acid position 127 in human CTLA4

ESP6500	Not present
dbSNP	Not present
1000G	Not present
ExAC	Not present
SIFT	Damaging (score 0)
Polyphen‐2	Probably damaging (score 1)
MutPred	Deleterious (0.862 probability)
PredictSNP	Deleterious
PROVEAN	Deleterious (score −8.456)
PON‐P2	Pathogenic (0.888 probability)
Mutation Taster	Disease causing
MetaSNP	Disease causing (score 0.785)
I‐Mutant Suite	Large decrease in protein stability (ΔΔG = −1.18 kcal/mol)
IStable	Decrease in protein stability (PDB 1I8L, ΔΔG = −1.5299; PDB 3OSK, ΔΔG = −1.18 kcal/mol)

## Outcome and Follow‐up

For the next 3 years, the patient remained free of infection on IVIG replacement (0.6–0.8 g/kg) every 3–4 weeks, with a median IgG concentration of approximately 6 g/L. In October 2016, he underwent a transurethral resection of the prostate and soon afterward developed diarrhea and significant weight loss. He was again admitted to our hospital, but after extensive investigation, no infectious or immune‐mediated cause could be found. There was an excellent response to a short course of a higher dose of oral prednisolone (30 mg/day, tapered over the next 2 months to 5 mg/day). In February 2017, he was admitted to his local hospital with left‐sided epididymo‐orchitis and rapidly died from hospital‐acquired pneumonia.

## Discussion

We describe a novel CTLA‐4 mutation causing a quantitative defect in protein expression associated with an altered phenotype of regulatory T cells. Tyr127Cys affects a highly conserved amino acid located in the ligand‐binding domain, close to the MYPPPY CD80‐ and CD86‐binding motif (amino acid positions 133–139), which is considered critical for protein function 18.

Inhibition of CTLA‐4 has recently become important in the immunotherapy of cancer, a side effect of which is multisystem autoimmune disease 19. In health, functional Tregs control autoimmunity, using CD25 and constitutive CTLA‐4 expression for fully competent effector function 20. In our patient, Treg frequency was reduced and we surmise that the Treg function was hampered, as a consequence of the significant reduction in CD25 and CTLA‐4 expressions within this subset. The severe reduction in memory B cells is consistent with his humoral immunodeficiency. Of note, *Enteroccocus* infection has been described in patients with Treg mutations 21. The low‐dose steroid regimen may have affected in vitro proliferation, but the overall changes are consistent with a defect in T‐ and B‐cell regulation due to CTLA‐4 haploinsufficiency with autoimmunity (Evan's syndrome), lymphoproliferation (diarrhea with intestinal inflammation), and immunocompromise (severe infections).

CTLA‐4 heterozygosity poses formidable diagnostic and treatment challenges. Abatacept (a fusion protein of the extracellular domain of CTLA‐4 and human IgG1) was not considered in our patient as immunodeficiency, rather than autoimmunity, predominated in the course of his disease. Similarly, bone marrow transplantation (BMT), which has been performed primarily in those with severe refractory autoimmune disease or chronic life‐threatening infections 3, did not seem appropriate in our patient. In hindsight, even though our patient survived to adulthood, in view of previous life‐threatening infections, he may have benefited from allogeneic BMT. We went as far as donor HLA assessment, but there was no familial match, and in any case, he was not accepted into a recipient list.

This patient was heterozygous for a novel CTLA‐4 missense mutation, which was predicted to be damaging and to affect protein stability and/or function. A causal link between CTLA‐4 heterozygosity, immunodeficiency, and autoimmune disease is strongly suggested but not proven. The mutation found, its predicted damage, and recurrent life‐threatening infections account for the seriousness and uniqueness of his condition. Particularly in those patients in whom the severity of immunodeficiency surpasses the autoimmunity, making the correct therapeutic choices remains an ongoing challenge.

## Ethical Approval

The patient signed informed consent for genetic testing and case publication.

## Conflict of Interest

The authors have declared no conflicts of interest.

## Authorship

All authors: participated in manuscript writing (mw); MM‐F: overall responsibility for mw and patient care (pc); AA, FL, AVT, AL: pc, and CM: cytometry; IC: computational predictions; APH, SMH, and GU: genetic testing, computational predictions, and cytometry.
